# Berry contagious: Use of post-exposure prophylaxis to respond to an outbreak of hepatitis A associated with frozen imported berries in Canada

**DOI:** 10.14745/ccdr.v52i09a02

**Published:** 2026-09-03

**Authors:** Elizabeth Schutt, Jamie Borlang, Colette Gaulin, Meghan Griffin, Melissa Helferty, Christina Lee, Rachel McCormick, Philippe Raymond, April Hexemer

**Affiliations:** 1 Centre for Foodborne, Environmental and Zoonotic Infectious Diseases, Public Health Agency of Canada, Guelph, ON; 2 National Microbiology Laboratory, Public Health Agency of Canada, Winnipeg, MB; 3 Ministère de la Santé et des Services Sociaux, Québec City, QC; 4 Canadian Food Inspection Agency, Ottawa, ON; 5 Ontario Ministry of Health, Toronto, ON; 6 Public Health Ontario, Toronto, ON; 7 Canadian Food Inspection Agency, Saint-Hyacinthe, QC

**Keywords:** hepatitis A, outbreak, foodborne, epidemiology, frozen berries

## Abstract

**Background:**

In April 2016, the Public Health Agency of Canada received reports of three locally-acquired cases of hepatitis A in one province who reported consuming frozen berries sold by a common retail chain. Subsequently, cases of hepatitis A with frozen berry consumption in other provinces were reported, and all illness onsets were within an eight-week period.

**Objective:**

To conduct a national outbreak investigation with provincial and federal partners to identify the source, and determine appropriate public health measures.

**Methods:**

Epidemiological data were collected through case interviews and purchase history records were obtained from the retailer. Hepatitis A virus (HAV) genotyping and ribonucleic acid (RNA) fingerprinting identified outbreak-related cases. The food safety investigation included traceback, traceforward, and testing of the suspected product.

**Outcome:**

Of the 25 confirmed cases, 21 were genotyped, revealing a multi-strain outbreak: 16 were genotype 1B with identical RNA fingerprints; and five cases were genotype 1A with nearly identical RNA fingerprints. Epidemiologic evidence implicated a frozen berry blend (Product A). Hepatitis A virus was detected in a closed retail sample, an open sample from a case’s home and a blackberry input. Product A was recalled, and post-exposure prophylaxis was offered. More than 11,000 people were vaccinated by the retailer and public health units. In September 2016, a new cluster of hepatitis A cases matching the outbreak strain was identified; the index case had consumed recalled product.

**Conclusion:**

Rapid epidemiologic investigation, purchase history records, traceback evidence, retailer cooperation and collaboration between public health partners facilitated rapid identification and product recall.

## Introduction

Hepatitis A is a nationally notifiable disease in Canada, where endemicity is low and has been reported at a rate at or below one per 100,000 individuals since 2007 (1). It is not part of the regular vaccination schedule except in the province of Québec (2). From 2014 to 2018, reported hepatitis A infections in Canada were predominantly genotype 1A (59%) or genotype 1B (26%), with fewer cases identified than genotypes 3A (15%) and 2A (0.3%) (*personal communication, A. Andonov, 2019*).

In Canada, local and regional public health authorities investigate cases of hepatitis A and report to the respective province or territory who then report age, sex and case counts to the nationally notifiable diseases surveillance system. Timely notification of increases in hepatitis A, based on a five-year rolling average, are provided on a weekly basis through the National Enteric Surveillance Program (3) or through notification from provincial or territorial public health partners. Provincial laboratories are encouraged to submit all hepatitis A virus (HAV) specimens to the National Microbiology Laboratory (NML) for genotyping and ribonucleic acid (RNA) fingerprinting. The NML maintains an extensive molecular database of primarily locally-acquired HAV strains in Canada, enabling cluster detection and notification when identical RNA fingerprints are detected in two or more cases (1,2).

Foodborne outbreaks of hepatitis A in North America and Europe have been linked to fresh and frozen produce, including sun-dried tomatoes, green onions, frozen berries and pomegranate seeds (4). Notably, in 2012 and 2013, two outbreaks of hepatitis A in Europe associated with contaminated berries affected over 1,400 individuals (5). One of these outbreaks was linked to two similar, but genetically distinct, strains of HAV in epidemiologically-linked outbreak cases (6). Subsequent hepatitis A outbreaks associated with frozen berries have been reported in Europe (7–10).

Between March 31 and April 5, 2016, two local health units in a single Canadian province identified three locally-acquired cases of hepatitis A who reported consuming frozen berries purchased from a single retail chain. Subsequently, other provinces reported hepatitis A cases with frozen berry consumption and all illness onsets were within an eight-week period. A national outbreak investigation was initiated in collaboration with provincial and federal partners to identify a common source of the illnesses and determine appropriate public health measures. This retrospective outbreak report describes the epidemiological, microbiological and food safety findings of the investigation, and the associated public health response to Canada’s first known multi-strain outbreak of HAV associated with frozen imported berries.

## Methods

### Case definitions

A confirmed case was defined as a resident of or visitor to Canada with laboratory‐confirmed HAV infection with 1) genotype 1B and identical RNA fingerprint or 2) genotype 1A and identical RNA fingerprint or 3) genotype 1A with a single nucleotide difference to the RNA fingerprint (**Table 1**); and where infection was likely acquired in Canada with a date of onset or laboratory collection date on or after January 1, 2016.

**Table 1:** Nucleotide sequences of ribonucleic acid fingerprints associated with the hepatitis A virus genotypes 1B and 1A outbreak strains

Outbreak strain genotype	Nucleotide sequence
1B	GATTGCAAATTACAATCACTCTGATGAATATTTGTCCTTTAGTTGTTATTTGTCTGTTAC AGAACAATCAGAGTTTTATTTTCCCAGAGCTCCATTGAATTCAAATGCCATGTTATCCA CTGAATCAATGATGAGCAGAATTGCAGCTGGAGACTTGGAGTCATCAGTGGATGATC CCAGATCAGAGGAGGACAGAAGATTTGAGAGTCACATAGAATGTAGGAAGCCATAC AAAGAATTGAGATTAGAAGTTGGGAAACAAAGACTCAAGTATGCTCAGGAAGAATTG TCAAATGAAGTACTTCCACCTCCTAGGAAAATGAAGGGACTGTTTTCACAAGCCAAAA TTTCTCTTTTTTATACTGAGGAGCAT
1A	GATTGCAAATTACAATCATTCTGATGAATATTTGTCCTTTAGTTGTTATTTGTCTGTCAC AGAACAGTCAGAGTTCTATTTTCCTAGAGCTCCATTAAATTCAAATGCTATGCTGTCCA CTGAGTCTATGATGAGTAGAATTGCAGCTGGAGATTTGGAGTCATCGGTGGATGATC CTAGATCAGAGGAGGACAGAAGATTTGAGAGTCATATAGAATGTAGGAAACCATACA AAGAATTGAGATTGGAGGTTGGCAAACAAAGACTCAAATATGCTCAGGAAGAGTTGT CAAATGAAGTGCTTCCACCTCCTAGGAAAATGAAGGGGCTATTTTCACAAGCTAAGAT TTCTCTTTTTTATACTGAAGAGCAT

A probable case was defined as a resident of or visitor to Canada with laboratory‐confirmed HAV infection using the Immunoglobulin M (IgM) antibody to HAV test (and who had not been vaccinated against HAV in the four weeks before onset), who reported consumption of the implicated frozen berry product within their illness incubation period (15–50 days before symptom onset) with genotype information pending or unavailable, and where infection was likely acquired in Canada with a date of onset or laboratory collection date on or after January 1, 2016.

### Laboratory analysis

Clinical specimens were screened for IgM anti-HAV at local community or private laboratories, or at provincial public health laboratories. Since 2013, the Public Health Agency of Canada (PHAC) has recommended that all IgM anti-HAV reactive clinical specimens be forwarded to the NML for genotyping and RNA fingerprinting. A 373-nucleotide fragment in the VP1-2A junction region was amplified to determine genotype and analysed for RNA fingerprinting as previously described (11). Due to the small size of the VP1/2A junction region, the complete VP1 gene was amplified whenever possible to improve the phylogenetic analysis of closely related HAV strains (12).

Testing for the presence of HAV in food samples collected from retail, the Product A manufacturing facility, and from case homes was completed by the Canadian Food Inspection Agency (CFIA) in Saint-Hyacinthe, Québec. The viral RNA was extracted from frozen food samples using a magnetic silica bead-based method (13) and detected by reverse transcription-quantitative polymerase chain reaction (RT-qPCR) (14). The HAV strain LSH/S (VR-2266, ATCC, Gaithersburg, Maryland, United States) was used as RT-qPCR positive control. The assay used for HAV detection targets and amplifies a conserved region of the viral genome located in the non-structural cysteine proteinase and RNA polymerase junction (3C/3D region) (15). This short, conserved region is a preferred target for a detection assay; however, it does not allow for further HAV typing since genotype 1A and 1B differ by only one or two nucleotides from the positive amplicon sequence in that region (**Table 2**). Presumptive positive HAV RT-qPCR amplicons were cloned, and five bacterial colonies per sample were selected for amplification and sequenced by Sanger sequencing (16) to confirm the results; however, food products that tested positive for HAV were neither further typed nor sequenced.

**Table 2:** Amplicon and reference sequences of the hepatitis A virus 3C/3D region used for detection in Product A and frozen blackberry input

Description	Sample and reference identification number	Amplicon and reference sequences^a^
NCBI reference genotype 1B	M16632	GGTCTCCAAAACGCTTTTTAGAAAGAGTCCCATTTATCATCACATTGATAAAACCATGATT
NCBI reference genotype 1A	Q425480	A.................................C....C.....................
Reference RT-qPCR control	AH003953	A.................................C.........................C
Food Product A	VI_020_2	A.................................C..........................
	VI_0042_001	A.................................C...............R..........
Frozen blackberry input	VI-0031-5	A.................................C..........................

Abbreviations: NCBI, National Center for Biotechnology Information; RT-qPCR, reverse transcription-quantitative polymerase chain reaction

^a^ R=A or G

### Epidemiological investigation

Cases were interviewed by local health authorities using either a routine provincial hepatitis A questionnaire or the national hepatitis A hypothesis generating questionnaire. All questionnaires were collected and analyzed centrally at PHAC to identify common exposures. Consent was requested from cases who reported consuming the suspected food product to obtain their individual purchase history from the relevant retail chain. Since Product A was a frozen product with a two-year shelf life, purchase histories of up to six months were requested. The information was used to confirm purchase of Product A in the weeks/months prior to illness onset.

### Data analysis

Descriptive analyses were conducted to describe cases by case definition category (confirmed or probable), HAV sequence, age, sex, hospitalization, frozen berry exposure and symptom onset date.

### Food safety investigation

Food samples from case homes were collected by local investigators, and the CFIA collected five closed food samples of the item of interest, Product A, from different retail locations. The CFIA conducted traceback, traceforward and food safety investigation activities related to Product A. The input import dates, and the Product A purchase dates captured in the case purchase histories were used to guide the Product A sampling plan at the manufacturing facility. To identify other potentially contaminated products, nine different lots of inputs (three blackberry, two cherry, four strawberry) and four different lots of finished products (retention samples) were collected by CFIA at the manufacturing facility.

## Results

### Case characteristics

Twenty‐five lab‐confirmed cases of hepatitis A were identified across three provinces in eastern Canada (**Figure 1**), where 21 cases met the confirmed case definition and four were classified as probable. Symptom onset dates ranged from February 26 to May 16, 2016. The median age of cases was 48 years (range: 10–70 years), and 56% were male (n=14/25). Ten cases (42% or 10/24) were hospitalized and no deaths were reported.

**Figure 1:**
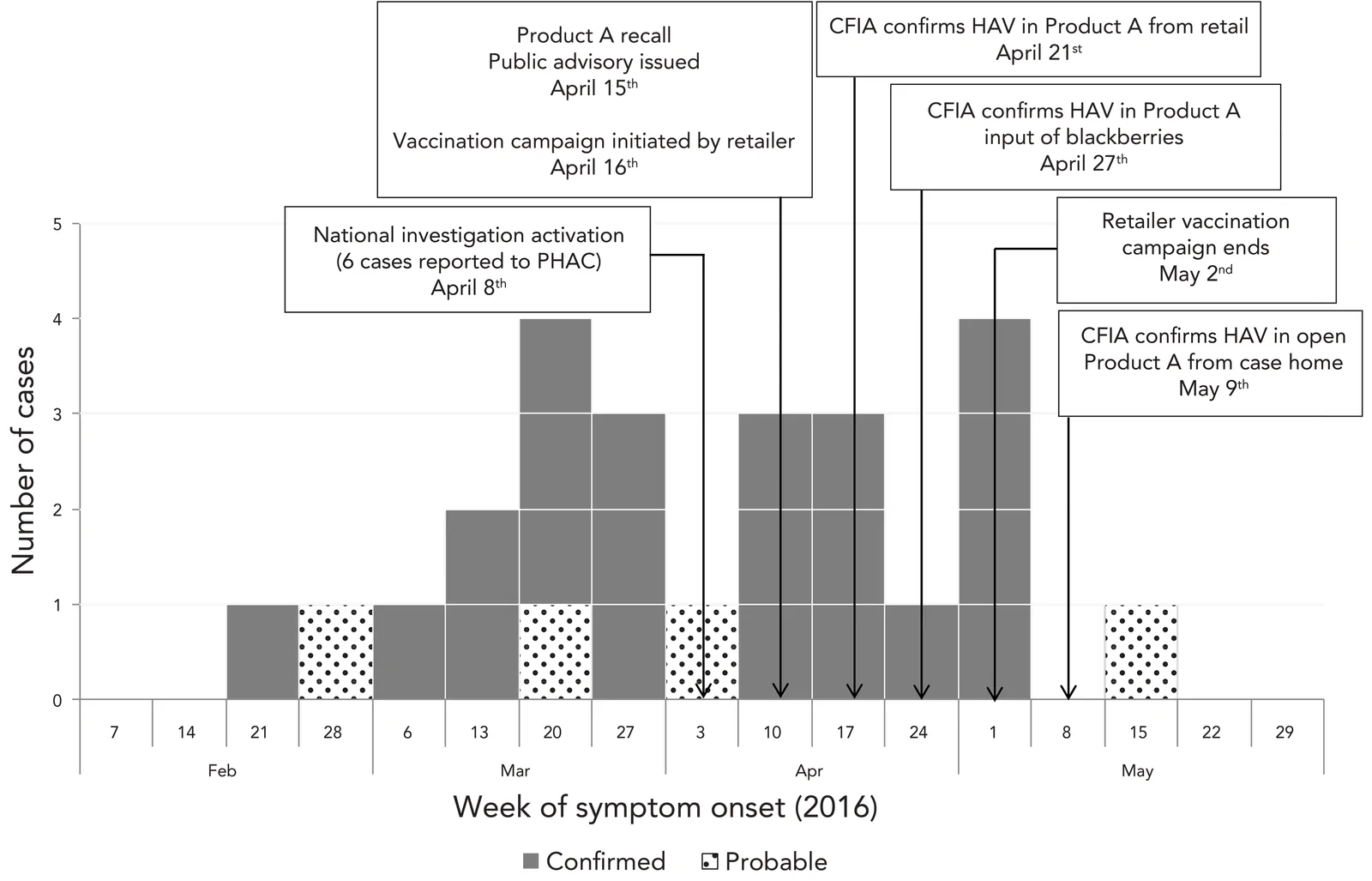
Distribution of confirmed and probable hepatitis A cases (n=25) by symptom onset and timeline of significant events, February 26–May 16, 2016^a^ Abbreviations: CFIA, Canadian Food Inspection Agency; HAV, hepatitis A virus; PHAC, Public Health Agency of Canada ^a^ Each box denotes a case

### Laboratory analysis

Genotyping and RNA fingerprinting were completed for 21 of the 25 outbreak cases. Sixteen cases were genotype 1B with identical RNA fingerprints, and five cases were genotype 1A with an identical RNA fingerprint match or an RNA fingerprint that differed by a single nucleotide. Genotype was not determined for four cases; these isolates were either not forwarded to the NML for genotyping, could not be typed or were PCR-negative.

### Epidemiological investigation

Twenty-three of 25 outbreak cases (92%) reported consuming or probably consuming Product A during their incubation period. The remaining two cases did not report consuming Product A but matched the primary 1B outbreak strain. Given HAV’s long incubation period (15–50 days), cases may not have recalled consuming Product A or may have unknowingly consumed the product outside the home (e.g., at a social gathering, restaurant or as a free sample offered by the retailer).

Thirteen purchase histories were retrieved from the 23 cases that confirmed shopping at the retail chain where Product A was sold. Product A was identified in 12 of the 13 retrieved purchase histories; where eight cases were HAV genotype 1B, two cases were HAV genotype 1A and two cases’ HAV genotype was not determined. Product A was not identified in the purchase history of the remaining case (HAV genotype 1A), due to a lost membership card, but the case verbally confirmed the purchase. Additionally, one case (HAV genotype 1B) confirmed consumption of the product at a friend’s home. Purchase dates of Product A were obtained from the friend, but no purchase history was retrieved.

### Food safety investigation

Product A was a mixture of frozen strawberries, blackberries, cherries, blueberries and raspberries sold at a single retail chain across five provinces in Eastern Canada. The individual berry inputs for this product were sourced from various suppliers in multiple countries and the final berry blend product was packaged at the manufacturer in Eastern Canada. Strawberries came from suppliers in Mexico and Turkey, blackberries from suppliers in Serbia and Bulgaria, cherries from suppliers in Turkey, blueberries from multiple suppliers in Argentina and Chile and raspberries from suppliers in Chile, Serbia and Bulgaria. Inputs from South America were not investigated as the predominant outbreak strain, genotype 1B, is more prevalent in eastern Europe and Mediterranean countries (17). Inputs from the previous six months were considered; older inputs were known to be unavailable at retail as finished product during the timeframe of interest.

One closed sample of Product A (best before date: March 15, 2018) from retail was positive for the presence of HAV. Of the 13 input ingredients and retention samples tested, one was positive for HAV: an input of blackberries imported from Bulgaria in February 2016, used only in Product A (best before date: March 15, 2018). There were no other Canadian importers of blackberries from this same Bulgarian exporter; however, Product A (best before date: March 15, 2018) contained four additional lots of blackberries, all from Bulgaria. These four additional lots were also used to make Product A (best before date: December 9, 2017), and a retention sample tested negative for HAV. One opened sample of Product A (best before date: February 2, 2018) from a case home was also positive for the presence of HAV. The sequencing results of the positive open and closed sample of Product A and the frozen blackberry input are shown in Table 2.

### Public health measures

A voluntary product recall was issued by the manufacturer for Product A (best before date: up to and including March 15, 2018) on April 15, 2016, seven days after the outbreak investigation was initiated. Public Health Agency of Canada, CFIA, provincial and local health authorities issued public messaging alongside accompanying social media messaging regarding the outbreak, the Product A recall, links to the retailer’s website and post-exposure prophylaxis (PEP) initiatives. The retailer posted information on their website regarding the product recall, including advice to customers and information on vaccination clinics. A timeline illustrating illness onsets and when significant events in the outbreak investigation occurred is shown in Figure 1.

Following the recall notice, the retailer used its membership program to issue two automated phone calls to members that had purchased Product A. The first call advised the member to not to consume Product A due to the recall, and the second informed the member of immunization being offered to eligible individuals and directed members to contact their local public health unit for more information. As per the guidelines for prophylaxis administered early in the HAV incubation period (18), all individuals who had consumed Product A in the previous two weeks were eligible for PEP offered by public health units in provinces where Product A was distributed, and by nurses and pharmacists at the retail locations where the product was purchased. The vaccination campaign was initiated by the retailer on April 16, 2016, the day after the product was recalled, and concluded on May 2, 2016. Approximately 2,578 individuals were vaccinated at local health units in three provinces, and 9,405 individuals were vaccinated at the retailer clinics. Numbers of vaccinations provided by public health in two of the five provinces were not available.

## Discussion

This was Canada’s first known multi-strain outbreak of HAV associated with widely distributed frozen imported berries. Multi-strain outbreaks linked to frozen berries have been reported in the United States (19) and Northern Europe (6,20). A common element of the frozen berry products implicated in these multi-strain outbreaks is the inclusion of inputs from multiple countries, as well as potential contamination of inputs by sewage. In this investigation, strong epidemiological evidence implicated two HAV strains associated with Product A. All but two cases with the predominant 1B outbreak strain and all cases with the 1A outbreak strain reported consuming Product A, with no other common food exposures identified. Although lab confirmation of both outbreak strains in Product A was not possible due to limitations in HAV detection methods, the epidemiological link was strong. This investigation demonstrates that multi-strain outbreaks associated with frozen berries are possible. Investigators should consider the possibility of a multi-strain event, especially if strong epidemiological evidence supports the hypothesis.

Purchase histories associated with store membership records, including product names and purchase dates provided the necessary specificity for action by public health and food safety partners. Supermarket receipts, store membership or purchase history data has been used to identify common exposures and confirm purchase of products in hepatitis A outbreak investigations in Canada (11) and Europe (6,20). In this investigation, prompt collection of purchase history data enabled quick identification of Product A, which was removed from the market seven days after the start of the national investigation. The collection of detailed food history of early cases, a clearly identified product distributed to a single retailer, and timely availability of purchase history information, contributed to the success of this investigation. Public health investigators should consider obtaining consent for loyalty/membership card or purchase histories early in an investigation, as they have proven useful in both hypothesis generation and product confirmation (21,22).

As a result of the outbreak investigation findings and subsequent product recall, the retailer initiated an extensive vaccination campaign. The availability of pharmacies within most of the retailer locations, direct calls to members who purchased the recalled product, and willingness of the retailer allowed for the prompt provision of prophylaxis. The same retail chain used a similar approach in a 2013 outbreak of hepatitis A associated with frozen pomegranate arils, with 165 affected individuals (19). Retailer membership data were used to identify and contact customers who had purchased the implicated product(s) to offer PEP, and more than 10,000 affected customers were vaccinated through in-store clinics.

Although the retailer was able to offer vaccination, jurisdictions reported challenges, including availability of vaccine, public and inter-agency communications and determining leadership of the PEP campaign. Coordination was complicated by varying provincial recommendations and criteria to guide decision-making around vaccine eligibility. This outbreak highlighted uncertainty about the role of public health authorities in decision-making regarding PEP for HAV, when retailers have desire and capacity to provide vaccinations. As a result, a protocol to support PEP decision-making in multi-jurisdictional foodborne hepatitis A illness outbreaks was developed to outline the roles and responsibilities of stakeholders, including public health authorities and the retail industry. It has been adapted and published as Annex 2 in Ontario’s Foodborne Illness Outbreak Response Protocol (23).

Several months after the outbreak concluded, a cluster of nine HAV cases matching the primary outbreak strain by RNA fingerprinting were identified in an eastern Canadian province where the implicated product had been sold. The index case had no travel history and had consumed the recalled Product A, confirmed by membership card information. Inadequate product recall communication was cited by investigators as a possible factor, as some individuals may not have been aware or complied with the recall. Case interviews revealed that several adult cases in the second cluster had contact with children who attended the same daycare, with no other commonalities identified. Investigation of the eight cases associated with the daycare indicated secondary transmission, and PEP was used as a means to prevent tertiary transmission. One case with no connection to the daycare reported travel to Bulgaria during their exposure period, providing evidence that this outbreak strain was circulating in that country. This later cluster, involving both foodborne and person-to-person transmission in a daycare, underscores the importance of broad communication of public health advice related to recalled products, especially those with a long shelf life.

A study has shown that in model experiments, frozen storage for three months had limited effects on HAV survival in frozen berries (24). This was observed in this outbreak, and was well described in a previous HAV outbreak investigation where frozen strawberries consumed by cases in February and March 1997 were traced back and their date of processing was April–May 1996, almost a year prior to the outbreak (25). During this event, broad and timely public communications were necessary to ensure that all consumers, including those exposed through shared food or in-store samples, were notified of the recall and availability of PEP. The identification of the second outbreak reinforced the importance of PEP campaigns to prevent additional illness associated with widely distributed retail foods, especially those like Product A with a long shelf life, and given the ability of HAV to persist under freezing conditions.

## Conclusion

This investigation adds to the evidence that frozen imported berries can be a source of HAV for countries with low endemicity. Rapid detection by the local health unit and detailed epidemiologic and purchase history information resulted in the rapid identification and recall of Product A from the market. The public health action including the PEP campaign provided by the retailer in collaboration with public health authorities likely prevented further illnesses. The second outbreak associated with consumption of recalled product highlights the importance of broad consumer communications, as recall notices may not reach all consumers.
